# Self-Sampling for Human Papillomavirus Testing: Increased Cervical Cancer Screening Participation and Incorporation in International Screening Programs

**DOI:** 10.3389/fpubh.2018.00077

**Published:** 2018-04-09

**Authors:** Sarah Gupta, Christina Palmer, Elisabeth M. Bik, Juan P. Cardenas, Harold Nuñez, Laurens Kraal, Sara W. Bird, Jennie Bowers, Alison Smith, Nathaniel A. Walton, Audrey D. Goddard, Daniel E. Almonacid, Susan Zneimer, Jessica Richman, Zachary S. Apte

**Affiliations:** ^1^uBiome, San Francisco, CA, United States; ^2^University of California San Francisco, San Francisco, CA, United States

**Keywords:** human papillomavirus, cervical cancer, cancer screening, self-sampling, vaginal microbiome

## Abstract

In most industrialized countries, screening programs for cervical cancer have shifted from cytology (Pap smear or ThinPrep) alone on clinician-obtained samples to the addition of screening for human papillomavirus (HPV), its main causative agent. For HPV testing, self-sampling instead of clinician-sampling has proven to be equally accurate, in particular for assays that use nucleic acid amplification techniques. In addition, HPV testing of self-collected samples in combination with a follow-up Pap smear in case of a positive result is more effective in detecting precancerous lesions than a Pap smear alone. Self-sampling for HPV testing has already been adopted by some countries, while others have started trials to evaluate its incorporation into national cervical cancer screening programs. Self-sampling may result in more individuals willing to participate in cervical cancer screening, because it removes many of the barriers that prevent women, especially those in low socioeconomic and minority populations, from participating in regular screening programs. Several studies have shown that the majority of women who have been underscreened but who tested HPV-positive in a self-obtained sample will visit a clinic for follow-up diagnosis and management. In addition, a self-collected sample can also be used for vaginal microbiome analysis, which can provide additional information about HPV infection persistence as well as vaginal health in general.

## Introduction

Cervical cancer takes the lives of about 250,000 women worldwide each year (1–3). This statistic is even more tragic given the fact that most of these deaths could be prevented with proper screening for precancerous lesions or the presence of human papillomavirus (HPV) (4) followed with standard clinical interventions. HPV DNA can be detected in the vast majority of cervical cancer tissue, and thus, HPV is considered the principal etiologic agent of cervical cancer (5, 6). Of the over 170 HPV types known to date, only some are associated with cervical cancer; collectively, these are called high-risk HPV (hrHPV) types. The main carcinogenic hrHPV types are 16, 18, 31, 33, 35, 39, 45, 51, 52, 56, 58, and 59 (7, 8). In addition, closely related HPV types such as 26, 53, 66, 67, 68, 70, 73, and 82 have been listed as possibly carcinogenic. Of these, hrHPV types 16 and 18 are detected in the majority (~70%) of cervical cancer samples worldwide (9), and the detection of these HPV types is associated with a high probability of cancer development within 1 decade (10).

## Cervical Cancer Screening Programs

Given the limited HPV types that appear to be the etiologic agents of cervical cancer worldwide, cervical screening constitutes an unusually unique opportunity to examine the impact of resources and methodologies on cancer prevention programs (11). Because the vast majority of cervical cancer is preventable after the detection of precancerous lesions or the presence of hrHPV, many countries have national cervical cancer screening programs in place, in which women are invited to undergo an in-clinic exam with follow-up visits and treatment in case of a positive finding. In countries where cervical cancer screening programs have been implemented, the incidence and mortality of this disease has shown a dramatic decrease over the past 20 years (12). The majority of industrialized countries, including the United States (US), offer cervical cancer screening programs to women aged 21 years and older, where women are invited to visit their physician for a pelvic exam at regular intervals (13). Most of these tests involve a Pap smear (also called a Pap test), in which a physician obtains a cervical specimen for histological or cytological staining and analysis (14). The test collects cells from the transformation zone of the cervix, using a small spatula and a brush, analyzing them under the microscope in search of abnormal morphology (14). To classify lesions, there are several nomenclature systems. Two of the most widespread are the cervical intraepithelial neoplasia (CIN) scale and the Bethesda system (15–17). The first distinguishes histological lesions by the fraction of epithelium replaced by undifferentiated cells into mild dysplasia (CIN 1), moderate dysplasia (CIN 2), and severe dysplasia and carcinoma *in situ* (CIN 3) (15–17). The Bethesda system is a cytological classification that describes abnormal findings as negative for intraepithelial lesion and malignancy, atypical squamous cells of undetermined significance, low-grade squamous intraepithelial lesions (LSILs) or high-grade squamous intraepithelial lesions (HSILs) (15–17).

Because these classification systems are based on human evaluation *via* microscopic analysis, and because virtually all cervical cancers are caused by hrHPV (5, 6), it has been proposed that molecular assays detecting DNA or RNA hrHPV markers might provide a better assessment of cancer risk than cytology (11, 17). Several hrHPV assays have been marketed, including Qiagen’s hybrid capture signal-based Digene HC2 HPV assay, and several PCR amplification-based tests, such as the Cobas test by Roche and the Xpert HPV test from Cepheid. Testing for the presence of hrHPV has proven to be more sensitive for cervical cancer precursors than the Pap test (18). In a large Kaiser Permanente study involving over 1 million women, 3-year risks for CIN3 or worse (CIN3+) or cancer following an HPV-negative result were lower than those following a Pap-negative result, suggesting that testing for HPV is more predictive for the reduced 3-year risk of developing cervical cancer and, thus, a better strategy for cervical cancer screening than a Pap smear (19).

These results support the use of hrHPV DNA testing for primary cervical screening, leading to recommendations from the US, Australia, and Europe to implement HPV screening in nationwide programs (20–22). In the US, screening guidelines provided by the American College of Obstetricians and Gynecologists (23) and the U.S. Preventive Services Task Force (USPSTF) (24) recommend women visit their healthcare provider every 3–5 years, depending on age and risk factors, for a Pap smear, often with HPV co-testing. In September 2017, the USPSTF released new draft recommendations for average-risk women aged 30–65 years old, abandoning co-testing, but instead proposing either cervical cytology every 3 years or hrHPV testing alone every 5 years (25). In both scenarios, samples are obtained by a physician during a pelvic exam. For women in high-risk groups, such as those with HIV infection or a compromised immune system, more frequent screenings are recommended.

## Barriers to Cervical Cancer Screening

Although free or low-cost cervical cancer screening is available in the US for women aged 21–64, not all women respond to these invitations. About 20% of women in the US eligible for cervical cancer screening have not been tested within the recommended timeframe (26, 27). This means that at least one in every five women in the US in the eligible age range, a group of at least 14 million women (27), have not been screened according to health guidelines. Screening participation is especially low among particular ethnic and socioeconomic groups within the US, including low-income groups, recent immigrants, and Native American, Native Hawaiian, Hispanic, and Asian populations (26–30). Similar poor responses to invitations and reminders for cervical cancer screening have been found among certain population groups in other countries as well (31). These disparities are likely to contribute to the higher invasive cervical cancer incidence and mortality rates found among certain ethnic groups (30, 32).

Multiple types of barriers preventing the participation in cervical cancer screening programs have been identified. First, subjective patient experience can decrease participation rates in conventional physician-performed cervical cancer screening (33). Feelings of embarrassment and shame are often mentioned as reasons to not participate in cervical cancer screening (31, 33–35). Women, in particular those of certain sociocultural groups, often report reluctance to having a physician see and touch their genital area (33). Women who have been sexually abused or who have experienced intimate partner violence are often uncomfortable with a standard pelvic exam (36, 37). In addition, the experience of discomfort or pain at a past clinical visit can discourage women from visiting a health professional again (31, 35, 38).

Second, lack of understanding about the importance of HPV or cervical cancer screening or underestimation of the risk of disease can also interfere with patient compliance. A study among 12,058 Norwegian women aged 25–45 showed that screening rates were highest among women who were aware of the recommended screening interval for cervical cancer (39) and similar results were found in China (38) and the UK (33). In addition, a meta-analysis showed that cancer awareness education—either *via* printed material or face-to-face home visits—can increase the participation of women in screening programs (40).

Third, practical challenges or socioeconomic barriers may also hinder patient compliance with recommended screening guidelines. In a 2014 study in the Netherlands among 10,000 women who answered a questionnaire about why they had not participated in past cervical screenings, most women answered that they had forgotten to schedule an appointment; other practical reasons were that they were pregnant, breastfeeding, or undergoing fertility treatment (41). Underestimation of the time elapsed since the previous screening has been identified as another factor associated with non-attendance (42). In a study among First Nations women in Canada, women living in small rural communities indicated that the time it would take them to drive to clinic for a Pap smear provided a significant barrier to accessing care, because of the disruption to their daily lives and the resulting difficulties with transportation or child care services (43). In countries without nationwide health insurance (such as the US), access to free or low-cost cervical cancer screening is not always readily available for the uninsured. In a National Health Interview Survey in 2013, it was found that only 60.6% of uninsured women in the US were compliant with their recommended Pap smear versus 85.2% of insured women (44, 45). Even in countries with universal healthcare, such as Canada and the UK, low socioeconomic status was associated with a lower compliance with cervical cancer screening. In a Canadian study, women in the lowest income neighborhoods were half as likely to be screened (46). Data from 2012 to 2013 obtained by the Primary Care Trust from the UK Health and Social Care Information Center showed that women from the highest quintile of income deprivation had 4.9 percentage points less coverage for cervical screening than women from the lowest quintile (47).

The socioeconomic and sociocultural barriers described above prevent many women from complying with recommendations for cervical cancer screening. Not surprisingly, cervical cancer rates are higher in women who have not been screened according to the recommended guidelines (48), with cervical cancer mortality rates being the highest in underscreened populations (30, 32).

## Self-Sampling may Increase Cervical Cancer Screening Participation

Offering women the option to self-collect vaginal or cervical samples at home has been proposed as a means to increase participation in cervical cancer screening programs. Self-sampling reduces the potential financial and logistical burden for the patient, and allows for a greater initial sense of privacy and autonomy. A recent meta-analysis encompassing 37 studies with 18,516 women from 24 countries across five continents indicated strong acceptance of self-sampling and a preference for self-sampling over clinician sampling (49).

Studies from a range of countries, both on the national level and on specific socioeconomic groups, have shown that offering self-sampling can lead to increased participation rates in cervical cancer screening (Table 1). In two large studies in the Netherlands, each among over 25,000 women who had not responded to invitations and reminders for an in-clinic visit and Pap test, one-third of the women did return a self-sampling device when provided with the option (50, 51). In a study of over 3,000 Norwegian women, offering self-sampling materials instead of an invitation for a physician-sampling visit increased attendance to 33.4 from 23.2% (52). Similarly, in a study performed among 4,060 Swedish women who had not been screened in at least 6 years, 39% accepted an invitation for self-sampling and HPV testing, while only 9% accepted an invitation for a Pap smear (53). A smaller, but still significant increase in response rate was found in a study on over 8,000 women in Finland (54). Two studies among Swedish women who had missed two previous screening rounds found the response rate to be two to three times higher if self-testing was offered compared to a standard screening invitation (55, 56). A large study among over 14,000 Italian women showed that 11.9% responded to an invitation to undergo an in-clinic Pap smear and 12.0% sent in a sample after having to pick up a kit at a pharmacy, compared with 21.6% who sent in a sample after receiving a self-sampling kit in the mail (57). A randomized controlled trial among 3,000 non-responder women in London showed that sending HPV self-sampling kits to persistent non-responders resulted in a 2.27-fold increased participation rate in cervical cancer screening in comparison with sending an invitation to attend for cervical cytology (58). A similar UK study performed among 6,000 women in the Newcastle upon Tyne region had a 2.25-fold higher response rate (59), while offering self-sampling in large studies performed in France resulted in nearly twofold to fourfold higher attendance rates (60, 61). Participation rates among a group of 8,000 underscreened Australian women were much higher when self-sampling was offered (20.3%) than when a Pap-smear reminder was sent (6.0%) (62). An even more marked difference was obtained in a study of 7,650 women in Argentina, where 86% of women who were offered to self-collect responded for an HPV test, while only 20% of women who were invited to attend a health clinic responded, representing a fourfold increase in patient compliance (63).

**Table 1:** Summary of randomized controlled trial studies mentioned in this review comparing participation rates in underscreened women offered either the option to participate in conventional, clinician-performed cervical cancer screening or vaginal self-sampling.

Reference	Country	Control group	Self-sampling group	Response to Pap-test invitation (%)	Response to self-sampling invitation (%)	*p*	Relative risk
Gök et al. (50)	The Netherlands	281	27,792	16.6	27.5	<0.001	1.66*
Lazcano-Ponce et al. (68)	Mexico	12,731	9,371	86.8	98.1	<0.001	1.13*
Piana et al. (60)	France	4,934	4,400	7.2	26.4	<0.001	3.67*
Szarewski et al. (58)	United Kingdom	1,500	1,500	4.5	10.2	<0.001	2.27*
Virtanen et al. (54)	Finland	6,302	2,397	25.9	31.5	<0.001	1.21
Wikström et al. (53)	Sweden	2,060	2,000	9	39	<0.001	4.33*
Gök et al. (51)	Netherlands	261	25,561	6.5	30.8	<0.001	4.73*
Darlin et al. (55)	Sweden	500	1,000	4.2	14.7	<0.001	3.50*
Sancho-Garnier et al. (69)	France	9,901	8,829	2	18.3	<0.001	9.15*
Broberg et al. (56)	Sweden	4,000	800	10.6	24.5	<0.001	2.32
Haguenoer et al. (61)	France	1,999	1,999	11.7	22.5	<0.001	1.92*
Arrossi et al. (63)	Argentina	2,964	3,049	20.2	85.9	<0.001	4.02*
Cadman et al. (59)	United Kingdom	3,000	3,000	6.1	13.7	<0.001	2.25
Giorgi Rossi et al. (57)	Italy	5,012	4,516	11.9	21.6	<0.001	1.82
Enerly et al. (52)	Norway	2,593	800	23.2	33.4	<0.001	1.44
Sultana et al. (62)	Australia	1,020	7,140	6	20.3	<0.001	3.38*
Viviano et al. (67)	Switzerland	331	336	92.7	94.3	NS	1.02*

*NS, not significant*.

*Asterisks denote values calculated for this review*.

A systematic review regarding different interventions to increase patient screening for various types of cancer combined 7 European studies on cervical cancer screening (several of which are mentioned above) and showed that mailing a self-sampling device for HPV testing directly to the patient resulted in an average 2.37-fold higher population participation in non-responder women when compared with a reminder for in-clinic Pap testing (64). In a meta-analysis of 10 studies, 8 from Europe and 2 from North America, the average compliance of HPV self-collected testing was 2.14 times higher compared to an invitation for a Pap smear. It was concluded that HPV self-sampling significantly improves the participation of women in cervical cancer screening (65). A more recent meta-analysis of 16 studies found similar results, with about 2.3 times more participants responding to a self-sampling kit sent to their homes, compared to an invitation for a clinician-obtained specimen (66).

Self-sampling does not always result in higher participation rates, though, and results are very much dependent on the country or population in which the study took place (Table 1). In a study with 667 Swiss women, participation in women offered self-sampling was not higher than among those offered sampling by a clinician (67). However, this country does not have a national cervical cancer screening program, and participation in both arms of the study was much higher than in most other countries. A similarly high participation rate in both arms of the study was found in a study performed among women in Mexico of low socioeconomic status and might be driven by the fact that women were all visited at their home by a nurse (68).

Self-collection might be of particular benefit for women of certain socioeconomic groups. In a study of 20,000 women from low-income communities in France, where low compliance with recommended Pap smear screening leads to 3,000 new cases of cervical cancer and 1,000 deaths each year, only 2% of women underwent Pap testing, while 18.3% of women responded to an invitation for a self-collected specimen for HPV testing (69). A study involving 346 women from underserved rural areas of Northern Greece, of whom only 17.1% had been regularly participating in Pap smear screening, found that 100% were willing to self-sample, with 90% willing to self-sample regularly if this option was available (70). First Nations women in Canada have a sixfold higher incidence of cervical cancer due to lower participation rates in cervical cancer programs; in a pilot program among 49 First Nations women, self-sampling was well received and the quality of samples was excellent (71). A second, larger study involving 834 First Nations women found a 1.3 higher response rate for self-sampling (72). In a study led by the University of Michigan, 93% of women from an indigenous community in Guatemala were willing to obtain a self-collected vaginal specimen, 88% provided a sample, and 79% found the test comfortable (73).

## Women Prefer Self-Sampling Over Sampling by a Healthcare Professional

Women participating in self-sampling trials for cervical cancer screening reported a positive experience. In a crossover trial in Hong Kong of self-sampling before undergoing a Pap smear, versus undergoing the Pap smear first, most women preferred self-sampling—in particular among women without previous experience of Pap smears. It was estimated that introducing self-sampling could increase participation rates of cervical cancer screening by 6.5% (74). In follow-up interviews with the First Nations study participants described above, many women stated that self-sampling removed key logistical barriers related to making a clinic visit, as well as removed the physical and emotional discomfort of a Pap test (43). A group of 746 Australian women who self-collected a vaginal sample and returned a questionnaire reported that the home-based test was less embarrassing, less uncomfortable, and more convenient than a clinician-performed Pap test (75). In a study among 1,069 woman in Mexico, women reported that the Pap test caused more discomfort, pain, and embarrassment than self-sampling (34). In a series of interviews with low-income indigenous Mexican women who were given self-sampling kits, most women identified the need to be screened for cervical cancer, but identified multiple barriers to making a clinic visit; the self-sampling kits were found less embarrassing and less painful than sampling by a healthcare professional (76). In a questionnaire of 3,049 women in Argentina who were invited to self-sample, most women preferred this method because it interfered much less with their daily responsibilities and was less time-consuming than a visit to a clinic (77). Similar results were found in a study in Santiago, Chile, where 86.5% of 1,254 women responded positively to an invitation to self-sample, and 91.6% of these reported self-sampling to be less uncomfortable than Pap testing (78). German women aged 20–30 years, who participated in a study to self-sample by cervicovaginal lavage rated the user-friendliness of the self-sampling method as easy (79). In a telephone survey of 199 low-income women in North Carolina who had not had a Pap test in 4 years, HPV self-tests delivered by mail were perceived to be trustworthy (80). However, in a recent study among 1,769 women presenting to two University of Washington clinics for routine cervical cancer screening, about 40% of participants were concerned that self-sampling might be inferior to clinician-collected samples, although both patients as well as physicians were supportive of the concept of self-sampling for HPV testing (81). In some studies, women reported that they were afraid to hurt themselves during sampling (76, 77, 82).

Together, these studies show higher participation rates in self-sampling than physician-performed Pap smear and HPV co-testing. In addition, most women reported positive experiences with HPV self-sampling, which could lead to improved patient compliance.

## Self-Collected Vaginal Samples are Comparable to Clinician-Collected Cervical Specimens for the Detection of HPV

Both patients as well as physicians have raised concerns about whether vaginal self-sampling is comparable to cervical clinician-sampling in detecting hrHPV. This agreement, often reported as kappa coefficient or concordance value, has been the topic of a large number of studies. Systematic reviews and meta-analyses from 2005 and 2007 found moderate to good HPV positivity agreement (kappa coefficient ranging from 0.24 to 0.96, overall sensitivity of 0.74 and specificity of 0.88) between these two sampling methods (83–85), while more recent studies have shown an excellent performance of HPV infection diagnosis on self-sampled vaginal specimens. In a 2014 meta-analysis lead by Arbyn and colleagues, data from 36 studies (on a total of 154,556 women) was used to assess the clinical accuracy of HPV detection on vaginal self-samples versus cervical clinical-collected samples to detect CIN2 or worse (CIN2+) (86). The sensitivity for HPV detection on self-samples was no different than clinical-collected samples for the detection of CIN3+. For cytology, using LSIL as the threshold, self-sampling was 14% more sensitive to detect CIN2+. For HPV detection, the authors found an overall 12% reduction in sensitivity for the detection of CIN2+ when compared to clinician-collected samples, but this reduced sensitivity was only associated with hybridization signal-based assays, such as used by the Digene HC2 assay. Of note, no reduced sensitivity was found if HPV screening was performed using amplification-based methods such as PCR. Overall, these results suggest that vaginal self-sampling is an equally good option for women who do not participate in screening programs involving physician-sampling, in particular if self-sampling is combined with DNA amplification, given its improved sensitivity compared against signal-based assays (86).

Other studies published after the meta-analysis by Arbyn and coworkers have confirmed agreement between vaginal self-obtained and cervical clinician-obtained samples for the detection of hrHPV types (Table 2). In a study among 1,845 Haitian women, HPV screening *via* self-collected vaginal swabs and clinician-obtained cervical swabs were 91.4% concordant (87). Using samples from 1,005 women in Papua New Guinea, 93.4% overall agreement was found between self-collected and clinician-collected samples using the PCR-based Xpert HPV test to detect hrHPV types (88). In a study among 194 women from Ghana, the overall HPV detection concordance of the two sampling techniques was 94.2% (89). A comparison between two vaginal self-sampling devices (*Evalyn* brush versus *Qvintip* collection device) and clinician sampling on 136 German women showed no significant differences in CIN2+ or CIN+ and specificity of hrHPV testing between self-sampling in comparison with clinician sampling; in addition, this same study showed agreement in the overall hrHPV detection rates between self-collected and clinician-collected specimens for both sampling devices, with a kappa of 0.82 for the *Evalyn* brush and a kappa of 0.78 for the *Qvintip* device (90). Comparing self-collected vaginal samples and clinician-collected cervical samples from 5,318 Scottish women showed that those two methods are equally sensitive and specific in detect cervical precancer stages (91). In a Swedish study among 218 women with abnormal cervical smears or with symptoms, the kappa value between clinician-obtained samples and vaginal self-sampling was 0.82 (92). Another study done in the Netherlands, including 2,049 women showed 96.8% hrHPV prevalence concordance between self-collected cervicovaginal samples and physician-taken cervical smears (93). Finally, a 2017 study among 232 Norwegian women with a diagnosis of cervical premalignant lesions or carcinoma found high agreement of hrHPV positivity between physician- and self-collected samples, depending on the sampling device and detection assay used (94). Together, these studies provide a robust support for the detection of HPV on self-obtained vaginal specimens, with results comparable to physician-collected cervical samples.

**Table 2:** Summary of recent studies mentioned in this review published after Arbyn et al. 2014 (86), comparing hrHPV detection in self-obtained vaginal samples to that in clinician-obtained cervical samples.

Reference	Country	Number of women	Collection device	HPV assay	Agreement HPV detection (%)	Kappa value	Relative sensitivity CIN detection (%)
Boggan et al. (87)	Haiti	1,845	Dacron brush (Qiagen)	digene HC2 (Qiagen)	91.4	0.73	0.90 (CIN2+)*
Jentschke et al. (90)	Germany	136	Evalyn brush (Rovers)	RealTime HPV (Abbott)	91.2	0.8	1.0 (CIN2+)*
Stanczuk et al. (91)	Scotland	5,318	Cobas swab (Roche)	Cobas 4800 (Roche)	NA	NA	0.97 (CIN2+)
Toliman et al. (88)	Papua New Guinea	1,005	Cytobrush	Xpert HPV (Cepheid)	93.4	0.74	NA
Asciutto et al. (92)	Sweden	218	Cobas swab (Roche)	Cobas 4800 (Roche)	93.9*	0.82	1.0 (HSIL)*
Ketelaars et al. (93)	Netherlands	2,049	Evalyn brush (Rovers)	Cobas 4800 (Roche)	96.8	NA	0.91 (CIN2+)
Leinonen et al. (94)	Norway	232	Evalyn brush (Rovers)	Xpert HPV (Cepheid)	91.4	0.66	0.97 (CIN3+)
Obiri-Yeboah et al. (89)	Ghana	194	careHPV brush (Qiagen)	careHPV (Qiagen)	94.2	0.88	NA

*NA, not available*.

*Asterisks denote values calculated for this review*.

## HPV Testing on Self-Collected Samples with a Follow-Up Pap Test is More Sensitive than a Pap Test Alone

Combining HPV self-sampling with a follow-up clinic visit and Pap smear to address a positive hrHPV result has proven more sensitive than a Pap smear alone. A meta-analysis by Snijders et al. concluded that hrHPV testing is at least as, if not more, sensitive for CIN2+ as histology on clinician-obtained specimens (82). Although hrHPV detection using self-sampling is less specific than clinician-collected samples exhibiting CIN2+ (i.e., hrHPV-positive specimens often show a less severe cytology), the increased sensitivity of self-sampling and HPV testing versus clinician-obtained Pap smear could potentially decrease morbidity and mortality associated with cervical cancer.

Other studies confirmed the high sensitivity of HPV testing from self-collected samples. For example, among a group of 615 women in Costa Rica, HPV testing of self-collected specimens was more sensitive for detecting CIN2+ than cytology. In addition, this study also showed that the proportion of women with initial normal baseline cytology that can develop CIN2+ during the follow-up is three times higher than the proportion of women with HPV-negative results (obtained from self-collection) that can develop CIN2+ later (95); this suggests that HPV screening may be more informative than cytology for predicting future cancer-related abnormalities. In a study performed among 2,000 Swedish women, women were sent an invitation for either self-sampling combined with an HPV test, or a Pap smear by a physician. Women who were HPV-positive after self-sampling were subsequently invited for further examination and histology. The odds ratio of finding histological CIN2 or CIN3 lesions with the self-sampling in comparison to the traditional Pap smear testing was 5.4 (53). Another study among 8,800 Swedish women found similar higher response rates among women who were offered self-testing and an odds ratio of CIN2 cytopathology detection of 2.0 (56). In addition, the use of self-sampling for HPV screening can also help to capture more HPV-affected individuals in the population. The chance of a woman having a positive cytology score was found to be more than 10 times higher in the case of a self-sampled positive hrHPV result, as found in an Italian study on 700 women (96). A large study including 28,000 women in the Netherlands found an odds ratio of 2.1 for the detection of CIN2+ lesions in women who had participated in self-sampling screening versus those that did not participate (50). Another study, comprising over 22,000 low-income women in Marseille, France, showed that detection of CIN2+ was higher among women offered self-sampling versus women who received an invitation for a Pap smear (69). In a study of 100,000 self-sampled Mexican women, the prevalence of hrHPV was 10.8%, and women with a positive hrHPV test had a relative risk of 15.7 for CIN2+ (97). Another large study including 13,140 Chinese women showed that HPV self-testing was more sensitive than cytology for the detection of CIN2+ (98).

The results of these studies, therefore, strongly suggest that the use of self-sampling in HPV detection with a follow-up Pap smear is a useful aid for the detection of abnormal cytologies, improving the detection when it is compared with the use of Pap smear alone.

## Women Who Self-Sample are Motivated to Undergo Clinician-Performed Follow-Up in Case of a Positive HPV Test

In addition to increasing patient participation and compliance, HPV self-sampling is also useful in motivating underscreened or never-screened patients to engage with their physician for ongoing screening and cervical health care. For example, in a trial reported by Broberg and coworkers (56), all nine women who tested positive for hrHPV attended an exam for cytology and colposcopy, suggesting that women with hesitations to undergo screening might be motivated to visit a healthcare provider following a positive self-sampling result. Another study conducted in Chile showed that 106 of 124 (85%) women who had not been screened in the previous 3 years but who were identified as HPV-positive after self-sampling, attended a later colposcopy (78). This number was even higher in the Norwegian study where 32 of 34 (94.1%) of the hrHPV-positive women in the self-sampling subgroup attended follow-up (52). In the study that included 7,000 underscreened Australian women, 106 of the 140 women (75.7%) who tested positive for hrHPV had colposcopy or cytology within 6 months (62), while in the Italian study mentioned above, 142 of the 168 women (84.5%) checked in at a clinic for follow-up examinations (57). The Dutch cohort involving 28,000 women mentioned above identified 757 HPV positive cases through self-sampling, 684 (90.4%) of whom presented for a follow-up with general practitioner (50), and similar high numbers were found in a second cohort (51). By contrast, the study among women in Marseille, France, had a self-sampling follow-up rate of only 41% (69), while in a more recent study performed in the same city, 52% of HPV-positive women went in for a follow-up Pap test. Based on the other studies mentioned above, however, most underscreened women who tested HPV-positive in a self-sampling trial were motivated to see their physician for follow-up care.

## More and More Countries are Accepting Self-Screening for HPV Testing

Although self-sampling for the detection of hrHPV types is not currently recommended as part of the standard of care in the US, it has already been implemented in many countries as a way to increase participation in cervical cancer screening and, thus, improve outcomes (99). The Netherlands was the first country to offer women the possibility to self-collect samples for HPV testing instead of going to a clinic for a Pap smear (93, 100, 101). In 2017, the National Cervical Screening Program in Australia switched to a recommended HPV screening every 5 years, with the ability to self-sample under medical/health care supervision (102). The Finnish Cancer Registry has also determined that self-sampling tests for HPV detection are reliable for cancer screening purposes (103, 104). Other countries have started trials with self-sampling to evaluate incorporation of this methodology in official national cervical cancer programs, including the UK (105), Norway (52), Denmark (106), and Switzerland (67). In addition, trials have started that incorporate self-sampling among particular populations with low screening attendance, such as the Maori in New Zealand (107), Haitian, Hispanic, and African-American women in South Florida (108), low-income women from North Carolina (109), and First Nations women in Canada (72). After the successful 2015 pilot study in Argentina by Arrossi et al. mentioned above (63), self-collection for HPV testing was scaled-up to include the complete Jujuy province (110). In addition, Romania will implement a new cervical screening system, including HPV detection and self-sampling in order to help to increase participation rates (111).

In the US, a recent randomized controlled trial was started in which underscreened women were offered either patient clinic reminders or the usual care plus home delivered hrHPV self-sampling kits (112). This trial is the first within the US to evaluate if self-screening could increase cervical cancer participation and be a part of future preventive care. Although the outcomes, such as predictive value to detect precancerous states, have not been reported yet, this trial is timely and an indication that the US might follow in the steps of other countries.

## The Role of Vaginal Microbiome Analysis in HPV Diagnosis and Monitoring

The associations between the vaginal microbiota and HPV acquisition, persistence, or progression is a growing area of research and potential treatment intervention. The vaginal microbiota may contribute to delayed HPV clearance, the triggering of carcinogenic pathways and, thus, cervical cancer risk (113, 114). Self-sampling for HPV with the addition of associated microorganisms may provide patients and providers with increasingly relevant and actionable clinical information.

In most women, the healthy vaginal microbiota is characterized by the dominance of one or two members of the *Lactobacillus* genus, Gram-positive bacteria that are thought to play a key role in the maintenance of a healthy vaginal environment (115, 116). Several microbial community states have been described, with the *Lactobacillus*-dominated states associated with health, and the more diverse states associated with conditions such as bacterial vaginosis (BV) (116–120). Specific vaginal microbiota signatures can also be seen during an HPV infection; including increased vaginal microbial diversity, decreased *Lactobacillus* spp. levels, and increased presence of specific microbes such as *Sneathia* spp. or *Gardnerella vaginalis* (121–125). Certain *Lactobacillus* spp. may be protective, while other vaginal microorganisms may increase a woman’s risk of HPV infection and cervical cancer (114). In a study of 70 healthy women, the vaginal microbial diversity of HPV-positive women was higher than that of HPV-negative women, and *G. vaginalis* was found at a higher frequency in HPV-positive women (122). In a longitudinal study of 32 women, each self-collecting twice weekly for 16 weeks, microbiota dominated by certain *Lactobacillus* spp. were associated with the clearance of HPV levels, while communities with low *Lactobacillus* spp. and high *Atopobium* spp. had the slowest clearance rates (121). In a Korean twin cohort with 68 female twins, HPV positivity was associated with a lower proportion of *Lactobacillus* spp., a higher microbial diversity, and higher counts of *Sneathia* spp. (123). In a study on 60 women from Chicago, certain *Lactobacillus* spp. abundance was inversely associated with HPV detection (124). In another study of 65 women, HPV infection was associated with a more diverse microbiome and a lack of certain *Lactobacillus* spp. (125).

Higher diversity of the vaginal microbiome and lower levels of *Lactobacillus* (particularly *L. jensenii)* are also associated with HSIL as compared to LSIL (126). Additional associations with HSIL include higher levels of species of *Sneathia, Anaerococcus*, and *Peptostreptococcus* (126). Patients with cervical cancer have also been shown to have a vaginal microbiota dominated by certain cytokines and *Fusobacterium* (127).

The vaginal microbiome composition as found in BV is in particular associated with the presence or clearance of HPV. A meta-analysis covering 12 studies showed a positive correlation between BV and HPV infection (128). In addition, patients with persistent HPV infection showed a significantly higher prevalence of BV than patients with HPV clearance (129). Another study showed an association between cervical neoplasia (CIN2+) and the presence of BV (odds ratio: 3.90), providing additional support for the association between BV, HPV infection, and cervical cancer development (130).

The vaginal microbiome is an emerging treatment area; HPV self-sampling with vaginal microbial analysis can help provide patients with additional information related to HPV, cervical cancer, and their overall vaginal health. In addition to standard guidelines for monitoring and treatment of abnormal results, patients may also benefit from microbiome specific interventions, including probiotics, prebiotics, dietary suggestions, hygiene and sexual practices, and contraceptive management (113, 114).

## Discussion and Conclusion

There is an international consensus that participation in cervical cancer screening programs remains a key factor in improving patient outcomes. However, many individuals do not comply with standard screening guidelines, often for a combination of reasons. For example, poor patient compliance may be caused by lack of time for a clinical visit, embarrassment related to the pelvic exam, and/or previous discomfort or pain during a Pap smear (34, 75, 76). Sociocultural and socioeconomic barriers may also cause women to postpone or decline regular cervical cancer screening. The percentage of women who have not had a Pap smear according to health care guidelines is higher among certain minority populations, such as American Indians and Asians, as well as those who live below poverty level (26), while US cervical cancer mortality rates among black women are twice as high as for white women (131). The use of self-collection for vaginal specimens for hrHPV screening has the potential to improve patient access to care, lead to higher patient compliance than current cervical cancer screening programs, and thus impact cervical cancer detection rates (64, 66, 74).

High-risk HPV testing on self-collected specimens with subsequent follow-up visit to a physician and cytology on positive cases has also been shown to be more sensitive when compared to Pap smears taken by a health professional in detecting CIN2+ pathology (82). In addition, a negative HPV test is more predictive for a reduced 3-year risk of developing cervical cancer than a negative Pap smear (19). Therefore, screening for hrHPV through self-sampling with appropriate follow-up for positive results may potentially be more effective than routine Pap smears (132).

Despite the advantages, self-sampling may also present new challenges for patient care. For example, self-sampling could conceivably decrease the opportunities for direct contact between the patient and the clinician, contributing to the possibility of decreased follow-up, as well as the potential for over-testing. In addition, women will need clear instructions to prevent feelings of insecurity during sampling and fear of hurting themselves (77). Another potential downside of self-testing might be an increase in downstream demands on the health care system. Self-sampling without appropriate follow-up or clear instructions on how to interpret a positive result also has the potential to increase patient anxiety, especially given the likelihood of many HPV infections to clear spontaneously (133). In all of these cases, HPV education is important to ensure appropriate patient engagement (40). Moving forward, additional infrastructure and guidelines will be needed to support the use of HPV self-sampling; new processes are already in development in many countries currently implementing self-sampling as part of their national cervical cancer screening protocol, such as focusing on women who have not responded to repeated invitations and addressing anxieties (133–135).

Remaining gaps in this field of research are how to better reach women from underscreened communities, both for cervical cancer screening as well as for follow-up treatment (27, 30). In addition, there is limited knowledge on the logistical and financial implications of shifting from a national screening program organized through in-clinic visits to that involving a self-sampling step (61). Finally, little is known on the long-term effects of the relatively new HPV vaccines, which are likely to change HPV epidemiology (136). The current HPV assays, which focus on the detection of hrHPV types covered by the vaccines, might need to be expanded to cover additional strains (136).

An emerging area related to HPV screening is the role of vaginal microbiome analysis in detecting the presence of commensal and pathogenic bacteria that are positively or negatively associated with HPV infection. Self-sampling has the potential to encourage women to engage regularly with their physician for appropriate cervical cancer screening, while also providing unique insights into vaginal health. Recent developments in vaginal microbiome testing have now made detection of HPV and associated microorganisms readily accessible, providing additional information with the potential to complement and improve the diagnosis and control of HPV infection and cervical cancer.

With the USPSTF now proposing a shift in cervical cancer screening for average-risk women aged 30–65 to hrHPV testing alone every 5 years (without cervical cytology), and several countries already offering self-sampling and HPV testing as a replacement for conventional Pap smear visits, similar developments are likely to take place in other countries. Because of the positive experiences in increased screening rates and cervical cancer prevention in other countries, self-sampling may become an even more viable option for many women in the US. With appropriate patient education and access to follow-up, HPV self-sampling has the potential to improve participation in screening programs, to reduce socioeconomic barriers to care, to improve the subjective patient experience, and, ultimately, to further reduce the continued morbidity and mortality related to HPV infection and cervical cancer.

## Author Contributions

SG, CP, EB, JC, and HN interpreted data and wrote the text. EB, JC, and HN did literature searches. LK, SB, JB, AS, NW, AG, DA, SZ, JR, and ZA contributed to the study conception and design. All authors approved the final version of the manuscript.

## Conflict of Interest Statement

All authors of the manuscript are current or past employees of uBiome, Inc. and have received stock options as well as other compensation. uBiome, Inc. funded the study concept, literature review, writing of the paper, and decision to submit for publication.
